# Structural Characterization of Oligochitosan Elicitor from *Fusarium sambucinum* and Its Elicitation of Defensive Responses in *Zanthoxylum bungeanum*

**DOI:** 10.3390/ijms17122076

**Published:** 2016-12-10

**Authors:** Peiqin Li, Robert J. Linhardt, Zhimin Cao

**Affiliations:** 1 Department of Forest Pathology, College of Forestry, Northwest A&F University, Yangling 712100, Shaanxi, China; zmcao@nwsuaf.edu.cn; 2 Center for Biotechnology and Interdisciplinary Studies, Rensselaer Polytechnic Institute, Troy, NY 12180, USA; linhar@rpi.edu; 3 Department of Chemistry and Chemical Biology, Chemical and Biological Engineering, and Biology and Biomedical Engineering, Rensselaer Polytechnic Institute, Troy, NY 12180, USA

**Keywords:** structural characterization, fungal oligochitosan elicitor, *Fusarium sambucinum*, *Zanthoxylumbungeanum*, plant–pathogen interaction, defensive response

## Abstract

Oligosaccharide elicitors from pathogens have been shown to play major roles in host plant defense responses involving plant–pathogen chemoperception and interaction. In the present study, chitosan and oligochitosan were prepared from pathogen *Fusarium sambucinum*, and their effects on infection of *Zanthoxylum bungeanum* stems were investigated. Results showed that oligochitosan inhibited the infection of the pathogen, and that the oligochitosan fraction with a degree of polymerization (DP) between 5 and 6 showed the optimal effect. Oligochitosan DP5 was purified from fraction DP5-6 and was structurally characterized using electrospray ionization mass spectrometry, Fourier transform infrared spectroscopy, and nuclear magnetic resonance spectroscopy. Oligochitosan DP5 showed significant inhibition against the infection of the pathogenic fungi on host plant stems. An investigation of the mechanism underlying this effect showed that oligochitosan DP5 increased the activities of defensive enzymes and accumulation of phenolics in host *Z. bungeanum*. These results suggest that oligochitosan from pathogenic fungi can mediate the infection of host plants with a pathogen by acting as an elicitor that triggers the defense system of a plant. This information will be valuable for further exploration of the interactions between the pathogen *F. sambucinum* and host plant *Z. bungeanum*.

## 1. Introduction

Plants are exposed to numerous microorganisms, but they are resistant to many potential pathogens because of their constitutive or inducible resistance [1]. Inducible defense responses include plant cell wall reinforcement by the deposition of lignin, necrotic hypersensitive response, biosynthesis of phytoalexins and pathogenesis-related proteins, and enhancement of the activities of defensive enzymes, such as phenylalanine ammonia lyase (PAL), polyphenol oxidase (PPO), peroxidase (POD), chitinase (CHI) and glucanase [2,3,4]. The inducible resistance of a plant often accompanies infection with a pathogen or stimulation by elicitors [5,6]. Extensive research over the past several years has shown that elicitors from pathogens can trigger defense reactions in host plants. One well-described elicitor from a pathogen that induces the biosynthesis of phytoalexin is a glucan heptasaccharide from the cell walls of *Phytophthora sojae* in soybeans [7]. Oligogalacturonides originating from plant cells have also shown the ability to elicit plant defenses against infection by pests [8]. Functionally active β,1→3 glucan elicitors are released in vitro within two hours by synchronously germinating zoopores of *Phytophthora megasperma* f. sp. *Glycinea*, which is associated with germ tube formation [9]. To date, many different types of elicitors from pathogens and non-pathogens have been shown to possess components that promote active disease resistance in plants [10,11].

Fungal cell walls are a source of regulatory molecules that control plant defense and development processes [12]. Chitin is a major component in the cell wall of most higher fungi [13,14]. Chitin or chitosan can act as elicitors that activate defense responses in plants, and they can be released by fungal pathogens during their invasion of host plants [15,16]. In plant tissues that have been invaded by pathogenic fungi, there are chitinases that can degrade chitin into soluble oligochitosan, which can be perceived by plant cells and induce defensive responses [17]. Oligochitosan activity that elicits defense responses has been found in many plants such as tobacco, rapeseed, rice, and grapevines [18,19]. This activity depends primarily on the degrees of polymerization (DP) and acetylation (DA) [20]. It is obvious that oligochitosan originating from a fungal pathogen can act as an efficient regulator of plant–pathogen interactions to induce host plant defenses. It is valuable to understand how oligochitosan from pathogens regulates plant–pathogen interactions and how plants can differentiate between these signals for appropriate responses to stimuli.

*Fusarium sambucinum* is a common pathogen causing dry rot in plants. Dry rot significantly decreases crop yields, and especially yields of potatoes [21]. In studies by our research team, *F. sambucinum* was observed to cause dry rot of *Zanthoxylum bungeanum* stems [22,23,24]. *Z. bungeanum*, also called “Huajiao” in China, is an economically important crop harvested as a food seasoning [25,26]. Dry rot of *Z. bungeanum* has caused great losses in the main producing provinces of China, especially in Shaanxi Province [24]. To date, there have been no reports on the interaction of *Z. bungeanum* with *F. sambucinum*. The aims of the present study were to structurally characterize the most efficient oligochitosan elicitor prepared from pathogen *F. sambucinum* and to explore how oligochitosan induces defense responses in the host plant *Z. bungeanum*.

## 2. Results and Discussions

### 2.1. Isolation of Chitosan and Oligochitosan from Fusarium sambucinum

Crude chitosan (CCH, 15 g) was extracted from dry mycelia (35 g) of *F. sambucinum*, and 13 g of CCH was further deacetylated to obtain deacetyated chitosan (DCH, 9.5 g). A mixture of total oligochitosan (TOCH, 5.5 g) was prepared by acid hydrolysis of DCH (8.0 g), and 4.5 g TOCH was further purified using a Bio Gel-P2 chromatography column. Four main fractions, DP < 5 (900.5 mg), DP5–6 (886.8 mg), DP7–9 (752.5 mg), and DP > 9 (828.6 mg), were obtained. Fraction DP5–6 (300 mg) was further purified, and pure oligochitosan DP5 (30.5 mg) with a carbohydrate content of 98.3% was isolated. The carbohydrate purity was increased by Bio Gel-P2 purification.

### 2.2. Effects of Chitosan and Oligochitosan on the Infection of the Pathogen on Z. bungeanum Stems

**Figure 1:**
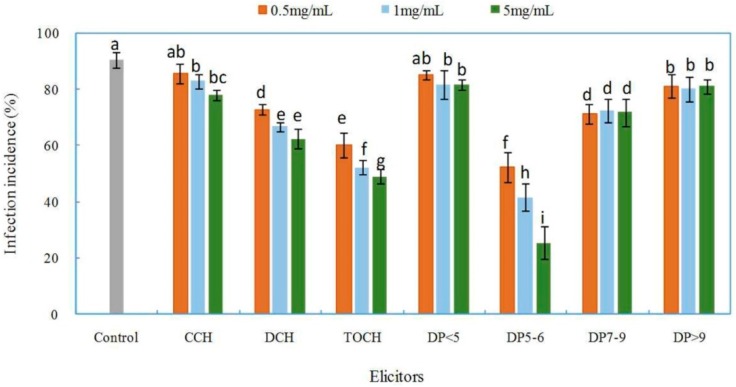
Effects of chitosan and oligochitosan elicitors from pathogen *Fusarium sambucinum* on infection of *Zanthoxylum bungeanum* stems. Different letters (a–i) indicate significant differences at a level of *p* < 0.05 of infection incidence among the treatments. CCH, crude chitosan; DCH, deacetyated chitosan; TOCH, total oligochitosan; DP, degree of polymerization.

The effects of chitosan and oligochitosan prepared from pathogen *F. sambucinum* on infection of *Z. bungeanum* stems were evaluated by determining the incidence of infection (Figure 1). Successful infection was confirmed by chlorosis and browning of bark at the inoculated site on *Z. bungeanum* stems. A lower incidence of infection indicates a stronger inhibitory effect of the applied compound on the infection of the pathogen on the plants [27,28]. There was an extremely high incidence of infection (90.5%) with the control sample, which indicated the high pathogenicity of pathogen (Figure 1). The incidence of infection incidence with all treatments, which were dramatically influenced by the forms and concentrations of elicitors, were lower than that in the control. DCH, the deacetylated product of CCH, showed greater inhibitory effects than those of CCH, with a lower infection incidence (62.2%) at a concentration of 5 mg/mL, indicating that the DA of chitosan influenced chitosan activity against pathogen infection. Chitosan with a low DA has been shown to better inhibit microbial cell growth, which might be attributable to the amine group in the C-2 position [29]. These results show that deacetylation of chitosan in the present study was necessary.

By hydrolysis of DCH, we obtained the mixture of TOCH. The incidence of infection was significantly different between plants treated with TOCH and those treated with DCH or CCH (Figure 1). TOCH reduced the incidence of infection, indicating that the molecular weight of chitosan affected its biological activity. By degradation of chitosan with a high molecular weight, oligochitosan with a low molecular weight and DP and excellent water solubility was obtained [30]. Oligochitosan has been shown to be more effective than chitosan in inhibiting the growth of various plant pathogenic fungi and eliciting various defense responses in plants, which can slow the development of plant diseases and directly or indirectly decrease disease severity [31,32,33,34]. TOCH was further purified to determine the effective fraction in TOCH that inhibited the infection of the pathogen. Four main oligochitosan fractions, DP < 5, DP5–6, DP7–9, and DP > 9, were obtained. The effects of the fractions on infection incidence depended significantly on their DPs (Figure 1). Fraction DP5–6 showed the greatest inhibition, and this inhibition was concentration dependent. When DP5–6 was applied at 5 mg/mL, the lowest incidence of infection (25.6%) was observed. The other three fractions did not show concentration-dependent inhibition of infection incidence. We speculate that DP5–6 might be the main effective component in TOCH that inhibits the infection of the pathogen on *Z. bungeanum* stems. Hence, fraction DP5–6 was further purified to characterize the structure and activity of pure oligochitosan.

### 2.3. Structural Analysis of DP5

Pure oligochitosan DP5 was isolated from fraction DP5–6, and its structure was analyzed by electrospray ionization mass spectrometry (ESI-MS), Fourier transform infrared spectroscopy (FT-IR), and nuclear magnetic resonance (NMR).

**Figure 2:**
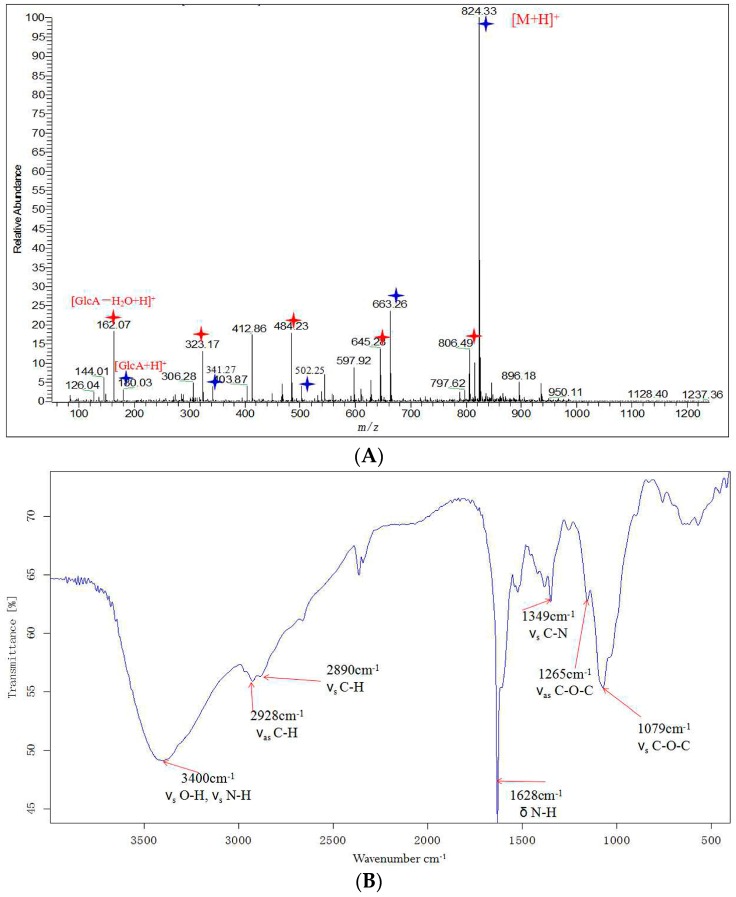
Positive ion electrospray ionization mass spectrometry (ESI-MS) spectrum (**A**); and Fourier transform infrared (FT-IR) spectrum (**B**) of oligochitosan DP5. The typical ion peaks for oligochitosan are indicated by blue and red star symbols in the ESI-MS spectrum. Blue stars represent the fragment ions 824.33, 663.26, 502.25 and 341.27 and 180.03 with one glucosamine residue decrease successively. Red stars represent the fragment ions 806.49, 645.28, 484.23, 323.17, and 162.07 with one glucosamine residue decrease successively. The important IR absorbances for oligochitosan are also shown in the FT-IR spectrum (red arrows).

By analyzing the ESI-MS spectrum, the molecular weight of DP5 was determined. An adduct ion peak [M + H]^+^ with *m*/*z* = 824.34 was observed (Figure 2A). The goal of our study was to isolate oligochitosan from *F. sambucinum*, and preparation and purification of an elicitor focused on oligochitosan. The structural unit of chitosan is glucosamine, which has the molecular formula C_6_H_13_O_5_N and a molecular weight of 179.14 [35]. When one glucosamine is linked with another to form an oligochitosan, one H_2_O molecule is lost; thus, the molecular weight of H_2_O needs to be subtracted from that of the resulting oligochitosan. A glucosamine residue (C_6_H_11_O_4_N) with a molecular weight of 161.19 Da was observed among the set of ions peaks with *m*/*z* at 824.33, 663.26, 502.25, 341.27, and 180.03, indicating that the oligochitosan had a DP of 5. Another set of ion peaks showed *m*/*z* at 806.49, 645.28, 484.23, 323.17, and 162.07 was also observed. Hence, this pure oligosaccharide was deduced to have a molecular weight of 823.33 and molecular formula of C_30_H_57_O_21_N_5_. This oligochitosan was named DP5.

FI-IR technology has been widely used to characterize oligochitosan [36]. The FT-IR of DP5 is shown in Figure 2B, and the important absorbance peaks are marked. In Figure 2B, “ν_s_” represents symmetric stretching vibration, “ν_as_” represents asymmetric stretching vibration, and “δ” represents bending vibration. The absorbance at 3400 cm^−1^ indicated the overlapping absorbance of hydroxyl groups (–OH) and amino group (–NH_2_) stretching vibrations, and the absorbance at 1628 cm^−1^ indicated the absorption of the bending vibration of the N–H bond. The absorbances at around 3400 cm^−1^ and 1628 cm^−1^, respectively, were characteristic for oligochitosan [37]. The absorbance at 1070 cm^−1^ was the absorption of the symmetric stretching vibration of band C–O–C. The characteristic absorbance for carbonyl bonds (C=O) of the acetamido groups of oligochitosan, which is in the range of 1900–1650 cm^−1^ [38], was not observed in the FI-IR spectrum of DP5. The lack of detectable absorbances for carbonyl bonds (C=O) demonstrated that deacetylation method used in this research was effective. The protocol for deacetylation of chitosan used in the present study, which was reported previously, almost completely deacetylated chitosan [39].

**Figure 3:**
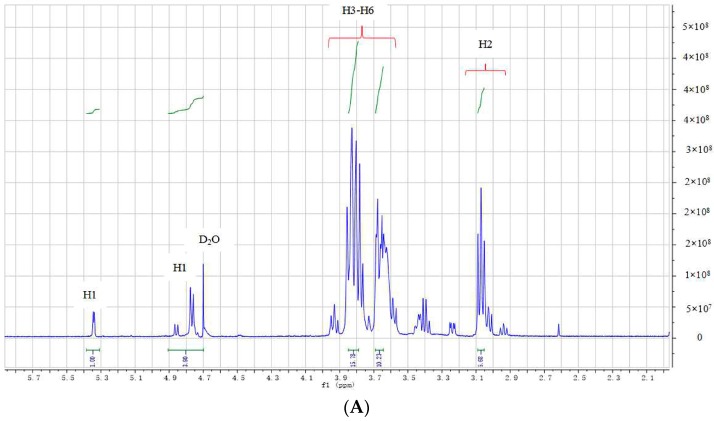

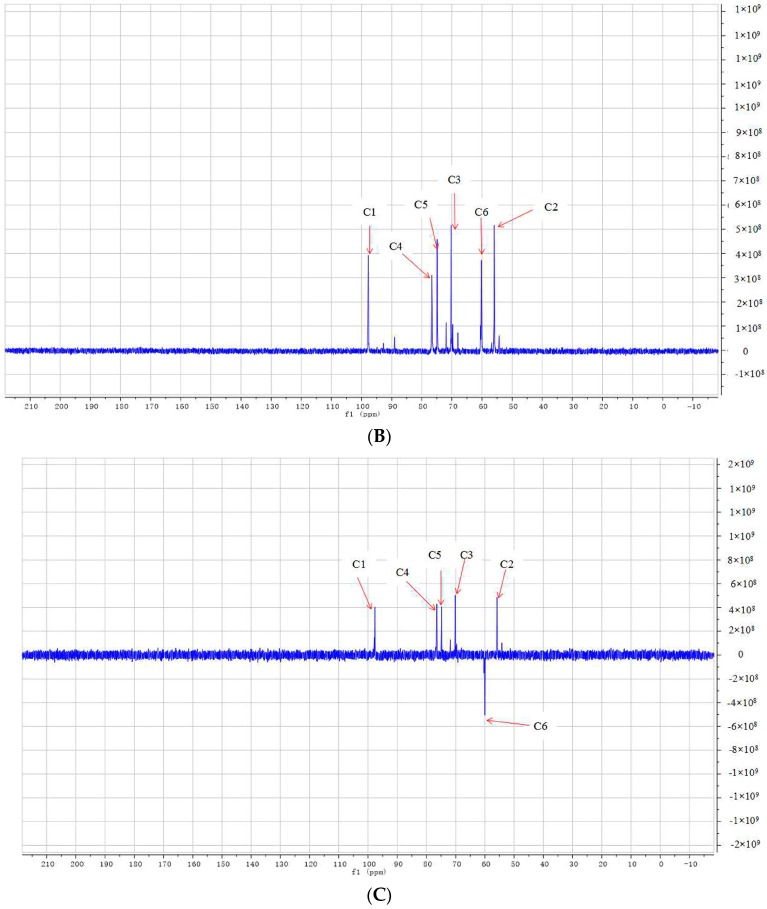
The spectra of: ^1^H-NMR (**A**); ^13^C-NMR (**B**); and DEPT-135 (**C**) of oligochitosan DP5. DP5 was dissolved in D_2_O at a concentration of 10 mg/mL. NMR, nuclear magnetic resonance; DEPT-135, distortionless enhancement by polarization transfer.

NMR spectroscopy is widely employed for the structural characterization of chitosan. The NMR spectra of DP5 were recorded in D_2_O. Based on analyses of ESI-MS and FT-IR spectra, the basic structural unit of oligochitosan DP5 was found to be glucosamine (GlcN). By comprehensive analyses, the spectra of ^1^H-NMR, ^13^C-NMR, distortionless enhancement by polarization transfer (DEPT-135), ^1^H-^1^H correlation spectroscopy (COSY), and rotating frame nuclear Overhauser effect spectroscopy (NOESY) ^1^H-^13^C heteronuclear single-quantum correlation spectroscopy (HSQC) and the chemical shifts of hydrogens and carbons in DP5 could be assigned. As presented in the ^1^H-NMR spectrum (Figure 3A), the chemical shift at δ 4.73 was attributable to the suppression peak of D_2_O by pre-saturation. The ^1^H-NMR spectrum showed a doublet peak at δ 4.77 that could be attributed to the anomeric hydrogen (H1) of GlcN connected to the anomeric carbon (C1), which indicated a β-configuration of the saccharide residues in DP5. The minor peak at δ 5.34 was the α form of the reducing end of the pentasaccharide. The signals in the range of δ 2.9 to δ 3.2 correlated with H2 from the GlcN unit (C2). Several signals in the range of δ 3.3 to δ 4.0 corresponded to the non-anomeric hydrogens H3-H6 in the sugar ring [38]. The minor peak at δ 1.96 might be attributable to a few residual *N*-acetyl groups [40], indicating the possible presence of a small amount of acetylated pentachitooligosaccharide. The minor peak at δ 2.55 might be attributable to impurities. The ^13^C-NMR spectrum of DP5 is presented in Figure 3B. The signal at δ 98.6 might represent anomeric carbon (C1), the signal at δ 56.7 might represent carbon 2 (C2) connected to NH_2_, and the signal at δ 60.0 might represent methylene carbon (C6). The carbon chemical shifts for C3, C4, and C5 of the sugar ring were δ 71.2, δ 78.2, and δ 75.1 respectively [41]. Six types of carbons in different chemical environments were observed in the DEPT-135 spectrum of DP5 (Figure 3C). The negative signal at δ 60.0 represented methylene (CH_2_), and the other five signals with the same numbers were for methine (CH). The DEPT-135 spectrum was consistent with the ^13^C-NMR spectrum of DP5. The other minor signals in the ^13^C-NMR spectrum might be attributable to impurities.

**Figure 4:**
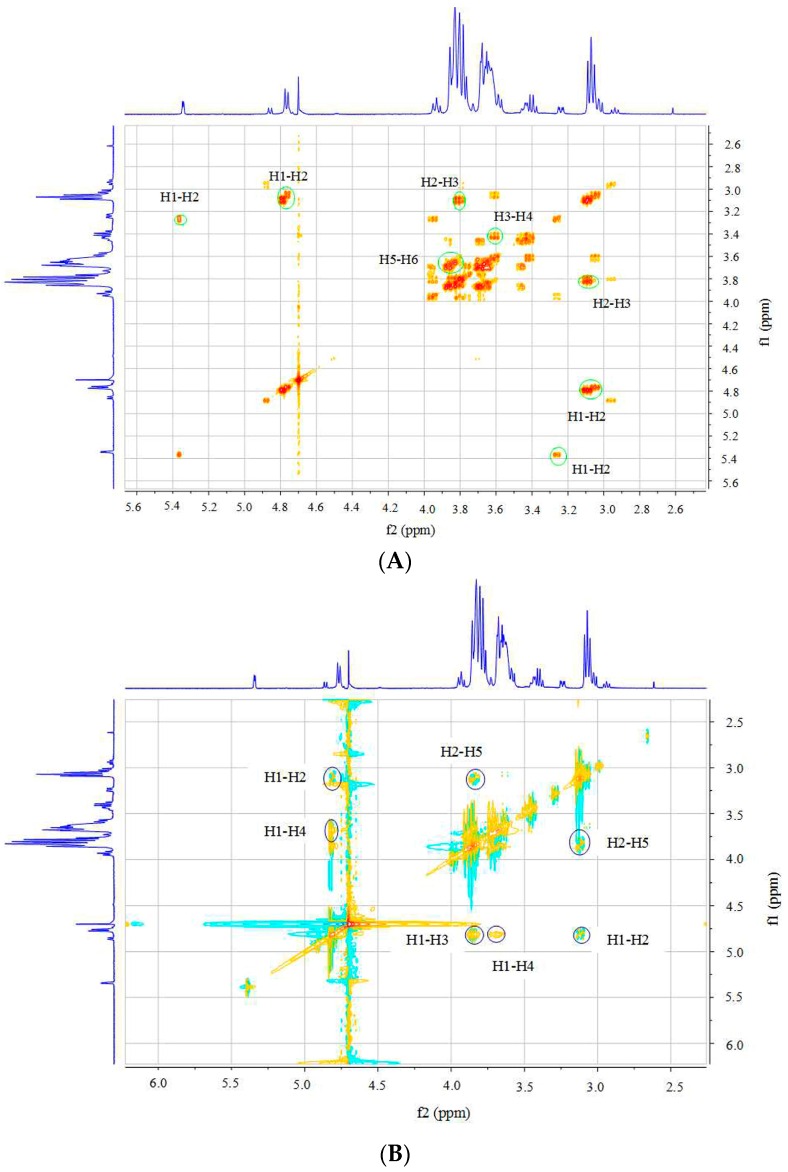

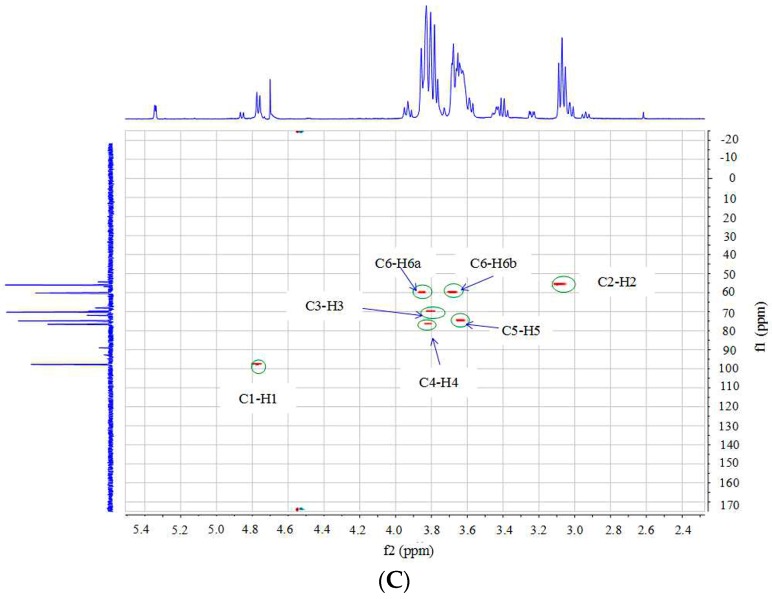
Two-dimensional nuclear magnetic resonance spectra of oligochitosan DP5: ^1^H-^1^H COSY (**A**); ROESY (**B**); and ^1^H-^13^C HSQC (**C**). Correlation peaks are marked on the spectra. COSY, correlation spectroscopy; ROESY, rotating frame nuclear Overhauser effect spectroscopy (NOESY); HSQC, heteronuclear single-quantum correlation spectroscopy.

Further assignment of chemical shifts for hydrogens and carbons were specifically presented from the two-dimensional (2D) spectra of ^1^H-^1^H COSY, NOESY, and ^1^H-^13^C HSQC (Figure 4). The cross peaks in the COSY spectrum (Figure 4A) provided information on the H1–H2 and H2–H3 correlations, which helped to assign the chemical shifts of H1 at δ 5.34 and δ 4.77, H2 at δ 3.05, and H3 at δ 3.80. Because of the overlap between the cross peaks for H3, H4, H5, and H6 in COSY, it was difficult to assign peaks. Data from the ^1^H-^13^C HSQC spectrum could be used to unambiguously assign the chemical shifts for hydrogens (H1–H6) and carbons (C1–C6) (Figure 4C). There were seven cross peak signals in the HSQC spectrum. In the GlcN sugar ring, each carbon was linked directly to one H, and five signals for the five C–H cross peaks were clearly observed in the HSQC spectrum, with all of these labeled in the HSQC by circles. The C at position C6 was linked to two H, and two cross peaks for C6–H6 could be easily recognized in the HSQC spectrum. The chemical shifts for H4 (δ 3.64), H5 (δ 3.83), H6a (δ 3.84), and H6b (δ 3.67) could be unambiguously assigned based on the HSQC spectrum. Analysis of the rotating frame Overhause effect spectroscopy (ROESY) (Figure 4B) of DP5 made it possible to determine the linkage position of GlcN residues. As shown in this ROESY spectrum, the signal for the H1–H4 cross peaks could be assigned, demonstrating the 1→4 linkage positions for GlcN residues.

**Figure 5:**
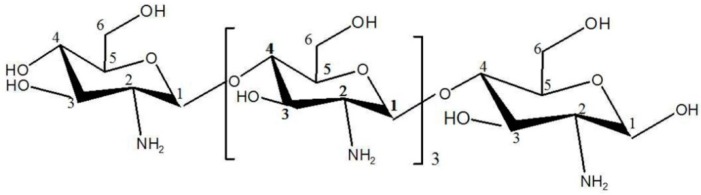
The structure of oligochitosan DP5, which is composed of five glucosamines.

Based on the above structural analyses, we conclude that oligochitosan DP5 has a molecular weight as 823 Da and is composed of five β-1→4 linked GlcN residues (Figure 5). This structure is consistent with previously reported structures of oligochitosan from fungi [42,43].

FT-IR and NMR have been used frequently to analyze the structure of chitosan, especially its degree of deacetylation [38,44]. In the present research, DP5 was almost completely deacetylated because there were no significant signals from acetyl groups in the FT-IR and NMR spectra. The overlapping chemical shifts of H3–H6 of the GlcN residue in the ^1^H-NMR spectrum makes it difficult to assign their respective chemical shifts precisely from only one-dimensional (1D) NMR spectra [45]. One-dimensional NMR and 2D-NMR were employed to completely characterize the structure of DP5. The oligochitosan DP5 isolated from *F. sambucinum* was not 100% pure because only Bio-Gel P2 column chromatography was used for its purification, because of limitations in experimental equipment. The carbohydrate content of DP5 was 94.3%, and some weak signals of impurities appeared in the ^1^H-NMR and ^13^C-NMR spectra. Although ^1^H-NMR has been widely used to analyze the structure of oligochitosans, most researchers focus on 1D-NMR and the chemical shifts of acetyl groups [45,46]. In the current research, 1D- and 2D-NMR were carried out to comprehensively characterize the structure of DP5 and to provide useful NMR data for future research.

### 2.4. Effects of Oligochitosan DP5 on the Infection of the Pathogen on Z. bungeanum Stems

**Figure 6:**
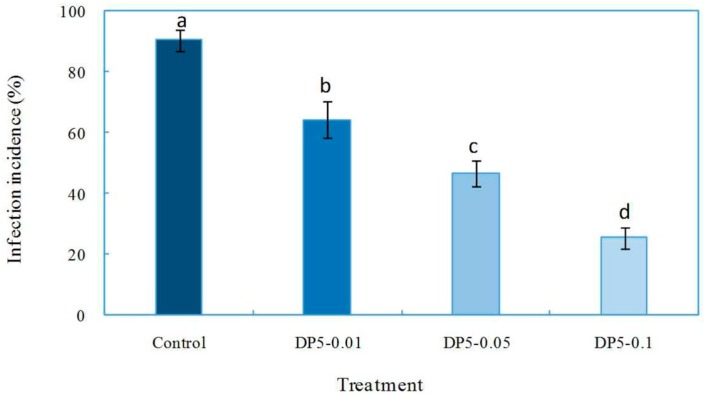
Inhibitory effects of oligochitosan DP5 on *Fusarium sambucinum* infection of *Zanthoxylum bungeanum* stems. Different letters (a–d) indicate significant differences at the level of *p* < 0.05 in the incidence of infection with various DP5 treatments. DP5–0.01, DP5–0.05, and DP5–0.1 indicate DP5 concentrations of 0.01, 0.05, and 0.1 mg/mL, respectively.

The effects of oligochitosan DP5 on *F. sambucinum* infection of *Z. bungeanum* stems were evaluated. Results showed that oligochitosan DP5 treatment at all three concentrations decreased the infection of the pathogen relative to that of the control (Figure 6). The incidence of infection in the control was 90.4%. When the concentration of DP5 was increased from 0.01 to 0.1 mg/mL, the incidence of infection decreased from 64.4% to 25.6%. DP5 was the major oligochitosan that was isolated and purified from fraction DP5–6. Fraction DP5–6 was the most effective crude fraction increasing the *F. sambucinum* infection incidence. Hence, DP5 may be considered the most efficient component of oligochitosan. The biological activity of oligochitosan is commonly recognized to depend on its molecular weight, DA, solubility, and the target organism in which it is tested [47,48]. Oligochitosan has been widely reported to induce defensive responses in plants [48,49].

In the present study, we systematically investigated the effects of chitosan and oligochitosan originating from pathogen fungi *F. sambucinum* on infection of *Z. bungeanum* stems with this pathogen. The effective oligochitosan monomer was obtained using a bioactivity-guided fractionation method, and its structure was specifically characterized using ESI-MS, FT-IR, and NMR technologies. The differential effects of oligochitosans on infection with pathogen were also observed based on differences in molecular weight. Oligochitosan showed stronger inhibition of the pathogen than chitosan, and the oligochitosan fraction with the DPs ranging from 5 to 6 exhibited the strongest anti-infective effect. Recently, it has been suggested that oligochitosans with high water solubility show the greatest biological activity [50]. Moreover, the effects of oligochitosans on elicitation of defense responses in plants are also related to their molecular weights, DPs, and DAs [48,51].

### 2.5. Induced Effects of Oligochitosan DP5 on Defensive Enzymes in Z. bungeanum

**Figure 7:**
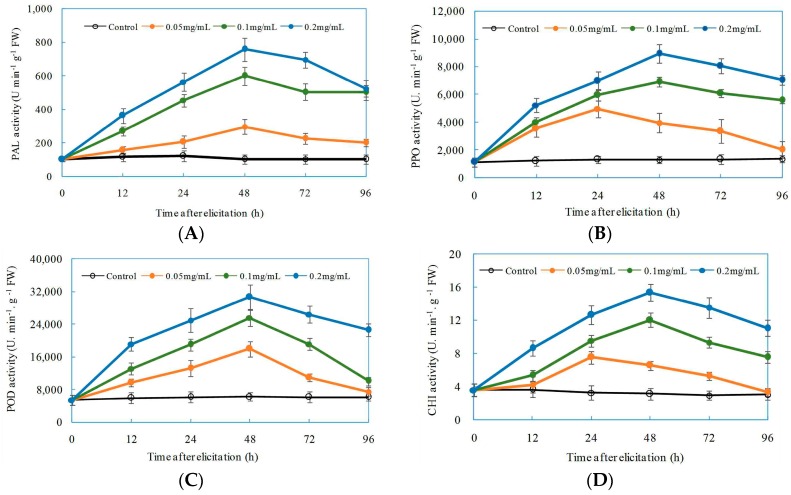
Effects of oligochitosan DP5 on the activities of defensive enzymes: phenylalanine ammonia lyase (PAL) (**A**); polyphenol oxidase (PPO) (**B**); peroxidase (POD) (**C**); and chitinase (CHI) (**D**) in *Zanthoxylum bungeanum*. The error bars represent standard deviations of the means from three independent samples. FW means fresh weight of plant material.

Inducible defensive enzymes PAL, PPO, POD, and CHI are involved in plant defenses against pathogen infection [52]. The application of oligochitosan DP5 in all treatments significantly enhanced defensive activities of all four enzymes in *Z. bungeanum* stems (Figure 7). The elicitation to defensive enzymes in *Z. bungeanum* stems was dependent on the concentrations of DP5 applied. As the concentration of DP5 increased from 0.05 mg/mL to 0.2 mg/mL, the activities of PAL, PPO, POD, and CHI significantly increased. As shown in Figure 7A, PAL activity increased gradually from 12 h after exposure, reached a maximum at 48 h, and then decreased slightly. The highest PAL activity was observed at 48 h when *Z. bungeanum* stems were treated with 0.2 mg/mL DP5; this activity was 7.4-fold greater than that of the control. The effect of DP5 on PPO activity in *Z. bungeanum* stems is presented in Figure 7B. Maximum PPO activity was observed at 48 h after application of 0.2 mg/mL DP5; this activity was 6.9-fold greater than that of the control. POD activities in *Z. bungeanum* stems after exposure to DP5 are displayed in Figure 7C. The greater POD activity was also induced by 0.2 mg/mL DP5 at 48 h; this activity was 4.9-fold greater than that of the control. Maximum CHI activity was obtained also at 48 h after *Z. bungeanum* stems were treated with 0.2 mg/mL DP5; this activity was 4.9-fold greater than that of the control (Figure 7D).

Oligochitosan DP5 strongly protected *Z. bungeanum* against dry rot. One mechanism related to this effect might be the enhancement of PAL, PPO, POD, and CHI activities in *Z. bungeanum* by oligochitosan DP5. PAL is a key enzyme in the first stage of phenylpropanoid metabolism that results in the biosynthesis of phenols, lignin, phytoalexins, and other compounds involved in localized plant disease resistance processes [53,54]. PPO in plants can oxidize monophenol, diphenol, or trihydric to their corresponding quinines, which can restrict the growth of plant pathogens [55]. The increase in POD activity elicited by oligochitosan was also related to an increase in resistance to pathogen infection [56]. POD participates into cell reinforcement and is involved in the final steps of lignin biosynthesis and cross-linking of cell wall proteins [57]. CHI has been intensively investigated and identified as a pathogenesis-related protein that functions in plant defense [58]. CHI is a hydrolytic enzyme that has direct antifungal activity by decomposing chitin in fungal cell walls [59]. Previous research has shown that chitosan can increase the expression of the genes for chitinase and peroxidase [60]. The elicitation of defensive enzyme in plants by oligochitosan has been reported to correlate with a reduction in disease incidence [35]. This induced resistance in plants might be the main mechanism by which oligochitosan inhibits the infection of the pathogen [61].

### 2.6. Effect of DP5 on the Production of Total Phenolics in Z. bungeanum

**Figure 8:**
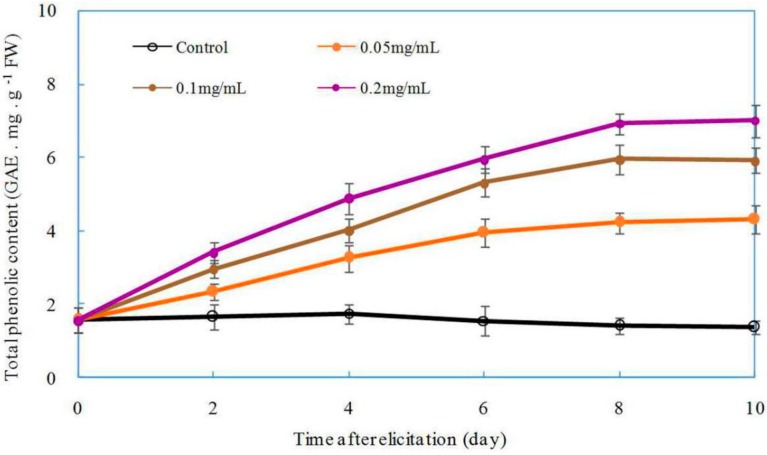
Effect of oligochitosan DP5 on the accumulation of total phenolics in *Zanthoxylum bungeanum*. Error bars represent standard deviations of the means from three independent samples. FW means fresh weight of plant material. GAE means Gallic acid equivalent.

The effect of oligochitosan on eliciting plant defense-related secondary metabolites such as pisatin, lignin, and phenolics has been widely studied in recent years [18,46]. We next investigated the effect of oligochitosan on the production of total phenolics in *Z. bungeanum*. The total phenolic content in *Z. bungeanum* increased significantly in response to oligochitosan DP5 as compared to that of the control (Figure 8). The effect of DP5 on the production of total phenolics correlated positively with the concentration of DP5 applied. The total phenolic content in *Z. bungeanum* twigs increased slightly from the moment of exposure to the 2nd day, rapidly and dramatically increased to its maximum on the 8th day, and then was maintained at a relatively stable level. When oligochitosan DP5 was applied at a concentration of 0.2 mg/mL, the maximum phenolic content was obtained in *Z. bungeanum* stems on the 8th day after treatment this content was 4.8-fold greater than that of the control. Because phenolic compounds in plants have been shown to possess antifungal activity [62,63], we conclude that the protective effect of oligochitosan DP5 on *Z. bungeanum* stems against dry rot is partially attributable to the accumulation of phenolic compounds.

Research on the fungal chitosan and its biological activities has increasingly been reported [64,65]. In the current study, we prepared chitosan and oligochitosan from the pathogenic fungus *F. sambucinum* and evaluated their effects on pathogen infection of the host plant *Z. bungeanum*. Results show that oligochitosan was more effective than chitosan in inhibiting the infection of the pathogen. The biological activities of oligochitosan correlated with its DP, DA, and solubility in neutral water [66]. The most effective concentration, DP, and DA of oligochitosan are different for different plant diseases, perhaps because of distinct mechanisms associated with specific plant–pathogen interactions [67,68]. In this study, oligochitosans with DPs between 5 and 6 exhibited the strongest inhibitory effects on the pathogen. Further purification yielded the oligochitosan monomer DP5 with an established structure. The completely deacetylated oligochitosan DP5 showed a significant inhibitory effect on pathogen infection of *Z. bungeanum* stems. We conclude that a high DA of oligochitosan from *F. sambucinum* is required to trigger the defense system of the host plant to prevent the infection of the pathogen.

In the present study, of the effects of oligochitosans on the infection of the pathogen on *Z. bungeanum* stems, we used the same stem for treatments with oligochitosans and the control. Oligochitosan treatment induced systemic resistance of the plant against the infection by the pathogen. Therefore, when oligochitosan was applied to the stem of *Z. bungeanum* using the present method, the results of the control might have been affected because of the systemic resistance. If we had used separate stems for the oligochitosan and control treatments, an even clearer effect of oligochitosan treatment may have been observed. In future studies, different methods to apply oligochitosan and to investigate the mechanisms underlying its affect should be employed.

Previous research has shown that defensive reactions of host plants are effectively induced by elicitors produced by pathogens [69,70,71]. The mechanisms through which oligochitosan elicits resistance of host plants against pathogen infection might be related to halting the growth of the pathogen directly, stimulating defensive enzyme activities, or promoting the synthesis of phytoalexins in plants [67,72]. The earliest discovered pathogen elicitors of plant resistance were heterogeneous branched β-1,3-glucans from the pathogen *Phytophthora megasperma* f. sp. *glycinea* (Pmg) [1]. These elicitors from Pmg very efficiently elicit glyceollin in soybean cotyledons [73]. Since the discovery of this elicitor, a series of studies on induced resistance in host plants elicited by oligosaccharides from host-specific pathogens were conducted, such as one on oligosaccharides from the cell walls of the soybean pathogen *Phytophthora sojae* that induced the synthesis of phytoalexin [73]. Thus, oligosaccharides from pathogens can induce plant resistance against diseases. It is a valuable to understand how plants can differentiate between these signals and appropriately respond to these stimuli to protect themselves from invading pests.

## 3. Materials and Methods

### 3.1. Materials and Reagents

The plant materials were the healthy stems of *Z. bungeanum* collected from the plant nursery of Northwest A&F University (Yangling, China). The dry rot pathogen of *Z. bungeanum* stems, *F. sambucinum*, was isolated in a previous study by our research team and preserved on slants of potato dextrose agar (PDA) medium [24]. Oligochitosan standards were purchased from Qindao BZ-OligoBiotech Co., Ltd., (Qiandao, China). All other chemicals were purchased from JieCheng Chemical and Glass Company (Yangling, China).

### 3.2. Cultivation of F. sambuciunum

Liquid fermentation cultivation of *F. sambucinum* was performed to obtain mycelia for the preparation of oligochitosan. *F. sambucinum* from the preservation slant was revived on PDA medium for 5 days at 25 °C and then transferred to new PDA plates and incubated at 25 °C for 7 days as the inoculation culture. Fermentation cultivation of *F. sambucinum* was conducted in 1-L Erlenmeyer flasks containing a liquid medium (300 mL) consisting of glucose (50 g/L), peptone (13 g/L), yeast extract (1 g/L), (NH_4_)_2_SO_4_ (5 g/L), NaCl (1 g/L), MgSO_4_·7H_2_O (0.5 g/L), and CaCl_2_ (0.5 g/L). The pH of the fermentation medium was adjusted to 6.0. All flasks were maintained on a rotary shaker at 150 rpm and 25 °C for 14 days. A total of 30 L of fermentation broth was obtained. The mycelia were separated from the fermentation broth by vacuum filtration using Whatman (Maidstone, UK) No. 4 filter paper and then washed several times with distilled water until a clear filtrate was observed. The mycelia were lyophilized and ground to powder with a high-power disintegrator. Finally, 35 g of mycelial powder from *F. sambucinum* was obtained for the extraction of chitosan.

### 3.3. Preparation of Chitosan and Oligochitosan from F. sambucinum Mycelia

The extraction of chitosan from fungal mycelia was carried out according to a previously reported method with some modifications [74]. The mycelial powder (35 g) was suspended in 4 L of 5% NaOH solution and autoclaved at 121 °C for 20 min. The alkali insoluble fraction (AIF) was collected by centrifugation at 12,000 rpm for 20 min and washed with distilled water three times and 95% ethanol twice. The AIF (20 g) was then treated with 1 L of 10% acetic acid solution at 95 °C for 8 h. The reaction suspension mixture was centrifuged at 12,000 rpm for 20 min. The supernatant solution was collected, its pH adjusted to 10 with NaOH (2 M), and then the supernatant was centrifuged again at 12,000 rpm for 15 min. The precipitated chitosan was washed with distilled water five times, 95% ethanol three times, and acetone twice, and then dried at 60 °C to a constant weight. The obtained crude chitosan (CCH) weighed 15 g.

Further deacetylation of chitosan was performed according to a previously reported protocol with slight modifications [37]. The crude chitosan (13 g) was suspended in 500 mL of NaOH (40%) solution and held at 110 °C for 1 h. The reaction mixture was then centrifuged at 12,000 rpm for 20 min. The precipitated chitosan was washed with distilled water several times and centrifuged until the pH of the supernatant was neutral. This procedure was repeated three times, resulting in almost completely deacetylated chitosan [39]. The chitosan finally obtained was dried at 60 °C to a constant weight. Finally, 9.5 g deacetylated chitosan (DCH) was obtained.

Oligochitosan was prepared by degradation of chitosan using hydrochloric acid according to a previously reported method with some modifications [75]. Deacetylated chitosan (8 g) was added to 1.2% acetic acid solution and stirred until the mixture was gelatinous. Hydrochloric acid (12 M) was then added to the gelatinous solution for a final concentration of 9 M hydrochloric acid. The reaction solution was held at 60 °C and 150 rpm for 24 h and then placed on ice to stop reaction. The reaction solution was vacuum filtered, and the filtrate collected and concentrated at 60 °C by rotary evaporator under vacuum to remove the HCl in the filtrate. The concentrated oligochitosan solution was mixed with 70% ethanol (1:3, *v*/*v*), held at 4 °C for 24 h, and then centrifuged at 8000 rpm for 20 min. The supernatant was collected and lyophilized to obtain total oligochitosan (TOCH, 5.5 g in weight).

### 3.4. Isolation and Purification of Oligochitosan

The total oligochitosan mixture (4.5 g) was fractionated by Bio-Gel P2 column chromatography (Bio-Rad Laboratories, Hercules, CA, USA) and eluted with distilled water with a flow rate of 0.5 mL/min. Each 5 mL of eluate was collected in a vial, lyophilized, and then subjected to thin-layer chromatography (TLC) detection using high performance silica gel plates with isopropanol-water-ammonium hydroxide (60:30:4, *v*/*v*) as the developing agent. The color-developing agent was composed of 2 g of diphenylamine, 2 mL of phenylamine, 10 mL of phosphoric acid (85%), and 100 mL of acetone [76]. By comparing the location of each fraction with those of standard oligochitosans by TLC, the fractions were combined according to their DP. Four main fractions were obtained and designated according to their DPs: DP < 5 (900.5 mg), DP5–6 (886.8 mg), DP7–9 (752.5 mg), and DP > 9 (828.6 mg).

After detecting the protective effects of these four fractions against dry rot on *Z. bungeanum* stems, the most effective fraction was chosen for further purification. In this study, the fraction DP5–6 showed the greatest protective activity. Hence, fraction DP5–6 (300 mg) was further fractionated by repeated Bio-Gel P2 chromatography with a flow rate of 0.2 mL/min. Finally, a pure oligochitosan fraction was obtained. By TLC detection and ESI-MS analysis, the DP of this oligochitosan was determined. The purified oligochitosan was named DP5 (30.5 mg).

The carbohydrate content of chitosan and each oligochitosan fraction was measured spectrophotometerically by the 3,5-dinitrosalicylic acid (DNS) method [77]. The DNS reagent contained a 1:1:1:1 volumetric mixture of 3,5-dinitrosalicylic acid (1%), Rochelle salt (40%), phenol (0.2%), and potassium bisulfate (0.5%), all dissolved in sodium hydroxide (1.5%). Chitosan or oligochitosan was first completely hydrolyzed by H_2_SO_4_ (98%) at 90 °C for 2 h, cooled on ice, and then neutralized with a NaOH solution (10 M). A certain volume of the obtained hydrolysate was used to react with DNS reagent in a boiling water bath for 5 min. After cooling to 20 °C, the absorbance of the reaction mixture was assayed at 540 nm, and the carbohydrate content was determined using glucosamine as a reference. 

### 3.5. Structural Identification of DP5

The molecular weight of DP5 was determined by ESI-MS on a Thermo Fisher LTQ Fleet mass spectrometer (ThermoFisher Scientific, Waltham, MA, USA). The infrared spectrum of DP5 was also assayed on a Bruker Fourier transform near-infrared spectrometer (FT-IR) (Bruker Corporation, Billerica, MA, USA) with wavelengths ranging from 400 to 4000 cm^−1^ in the solid state using potassium bromide pellets. For the NMR analysis, the DP5 sample (10 mg) was dissolved in D_2_O (99.98%). The NMR analysis of DP5 was carried out on a Bruker ADVANCE 500 MHz spectrometer at 70 °C with D_2_O suppression by pre-saturation. DEPT-135, COSY, ROESY, and HSQC were recorded using standard Bruker pulse sequences.

### 3.6. Effect of Chitosan and Oligochitosan on Pathogen Infection of Z. bungeanum Stems

Healthy uniform *Z. bungeanum* stems (diameter 2 cm, length 25 cm) were collected from the plant nursery of Northwest A&F University. The surfaces of stems were cleaned. Each stem included six round inoculation sites. The diameter of each inoculated site was about 0.5 cm, and the distance between any two inoculated sites was about 3 cm. Because the pathogen invades through a wound, we used an autoclaved dissecting needle to make seven minor stab wounds at each inoculation site. The inoculum was 7-day-old *F. sambucinum* mycelial cake with a diameter of 0.5 cm.

In this study, we investigated the effects of CCH, DCH, TOCH, fraction DP < 5, fraction DP5–6, fraction DP7–9, and fraction DP > 9 on infection of *Z. bungeanum* stems. The concentrations of each elicitor were 0.5, 1, and 5 mg/mL in sterile distilled water. Each elicitor solution was filtered through a sterile filter membrane (pore size, 0.45 µm). The treatment method involved wrapping the inoculated site with sterile cotton (length 2 cm, width 1 cm, thickness 0.2 cm) soaked in 1 mL of elicitor solution. Control experiments were carried out using sterile distilled water. Each stem was exposed to three replicates of each treatment and three controls, and each treatment was applied to 10 stems. In total, there were 30 inoculation sites for each treatment and 630 inoculation sites for the control. All of the stems were placed into a beaker containing enough water to maintain the normal physiological function of stems, and then they were transferred to a growth chamber at 25 °C with 12 h of illumination every day. After 7 days, symptoms of infection were monitored, and the inoculated sites of all stems successfully infected by *F. sambucinum* were counted. The infection incidence (%) was calculated as the percent of infected sites among total inoculated sites, which was taken as the indicator of the effects of oligochitosans on the inhibition of infection of the pathogen on *Z. bungeanum* stems. The experiment was conducted twice.

### 3.7. Effects of Oligochitosan DP5 on the Infection of the Pathogen on Z. bungeanum Stems

DP5 was the main pure oligochitosan isolated from the DP5–6 fraction showing effective inhibition of the pathogen on *Z. bungeanum* stems. Hence, we speculated that DP5 might be the effective component of fraction DP5–6. The inhibitory effects of DP5 of the pathogen on *Z. bungeanum* stems were further examined to verify our assumption. The concentrations of DP5 were 0.01, 0.05, and 0.1 mg/mL. Ten stems were used for this experiment. Hence, there were 30 inoculation sites for each treatment and 90 inoculation sites for the control. The experiment was conducted twice. The infection incidence (%) was calculated for each treatment. A lower infection incidence means the inhibitory effect of DP5 against the pathogen is stronger.

### 3.8. Elicitation of Defensive Enzymes in Z. bungeanum by Oligochitosan DP5

The activities of defensive enzymes, including PAL, PPO, POD, and CHI, in *Z. bungeanum* twigs treated with DP5 were assayed. Fresh healthy uniform twigs of *Z. bungeanum* from the current year (30-day-old plants) were used in the experiment. Each twig had an incision on its bottom to absorb DP5 solution and was placed in a 5-mL Eppendorf tube containing 1 mL of DP5 solution for approximately 5–7 h to ensure that all of the DP5 solution was absorbed by each twig. The concentrations of DP5 used in for treatment were 0.05, 0.1, and 0.2 mg/mL. Control twigs were treated with sterile distilled water. To maintain the normal physiological function of the twigs, they were placed in beakers containing a certain amount of autoclaved water. After that, they were transferred into a biological incubator at 25 °C under 12 h of illumination every day. The stems were collected separately at 12, 24, 48, 72, and 96 h after treatment and then used to extract crude defense-related enzymes. Each treatment was conducted in triplicate.

PAL activity was calculated by the production of trans-cinnamic acid converted from l-phenylalanine by PAL using spectrophotometry [54]. The fresh weight (FW) of each sample for PAL extraction was 0.5 g. The twigs were ground in 5 mL of pre-chilled borate buffer saline (0.05 mg/mL, pH 8.8) with 5.0 mmol/L β-mercaptoethanol. The extraction homogenate was centrifuged at 13,000 rpm at 4 °C for 20 min in a refrigerated centrifuge. The supernatant constituted the enzymatic crude extract, which was transferred to a 1.5 mL vial and stored at −20 °C. The reaction mixture for PAL activity contained 50 µL of BBS (pH 8.8), 50 µL of enzyme extract, and 100 µL of l-phenylalanine (0.2 mol/L). The reaction mixture was incubated at 40 °C for 1 h. After this, the reaction was stopped by the addition of 50 µL of HCl (2 mol/L). Absorbances were determined at 290 nm using a microplate reader. One PAL unit (U) was defined as a change of 0.01 OD per minute per gram of FW at 290 nm. PAL activity is presented in U·min^−1^·g^−1^ FW.

The crude PPO and POD enzymes were extracted from *Z. bungeanum* twigs using methods described in our previous report [76]. The crude enzymatic extraction was carried out by grinding treated twigs (0.5 g, FW) in 5 mL of pre-chilled 0.05 mol/L phosphate buffered saline (PBS, pH 6.8) extraction buffer with 1% polyvinyl polypyrrolidone (PVPP). The extraction mixture was centrifuged at 13,000 rpm at 4 °C for 20 min. The supernatants were transferred to new 1.5-mL vials, stored at −20 °C, and later used for measurements of PPO and POD activities.

PPO activity was measured spectrophotometrically as the change in absorbance at 398 nm for 2 min, based on our previous research [27]. Catechol was used as the substrate. A reaction volume of 200 µL included 100 µL of catechol (0.05 mol/L), 50 µL of crude enzymatic solution, and 50 µL of PBS (0.05 mol/L, pH 8.8). A change of 0.01 OD at 398 nm per minute per gram FW was considered one PPO unit (U). PPO activity is presented as U·min^−1^·g^−1^ FW.

POD activity was tested using guaiacol as the hydrogen donor [78]. The reaction mixture included 10 µL crude enzymatic solution, 25 µL guaiacol (1%), 25 µL H_2_O_2_ (1%), and 150 µL PBS (0.05 M, pH 8.8). The reaction for the POD assay was carried out at 37 °C for 10 min. The absorbance of the reaction mixture was assayed at 470 nm. A change of 0.01 OD at 470 nm per minute per gram FW was considered one POD unit (U). POD activity is presented as U·min^−1^·g^−1^ FW.

CHI extraction was conducted according to a previously reported method [79]. The treated twigs (0.5 g, FW) were sliced and then homogenized in 5 mL of chilled sodium acetate buffer (100 mM, pH 5.0) containing 5 mM β-mercaptoethanol and 1 mM ethylenediaminetetraacetic acid (EDTA). The homogenate was centrifuged at 13,000 rpm for 20 min at 4 °C, and the resulting supernatant was collected for CHI activity assay. CHI activity was determined using a previously described method with some modifications, including carboxymethylchitin-ramazol brilliant violet solution (2 mg/mL, CM-Chitin-RBV) as a substrate [80]. The reaction mixture included 50 µL of crude enzyme solution, 100 µL of CM-Chitin-RBV solution (2 mg/mL), and 50 µL of sodium acetate buffer (100 mM, pH 5.0), which was mixed and incubated for 2h at 37 °C. After incubation, the reaction was terminated by adding 50 µL of HCl (1.0 M) to precipitate the unhydrolyzed polymeric substrate molecule CM-Chitin-RBV. The reaction mixture was cooled on ice for 10 min and then centrifuged at 13,000 rpm for 5 min. The supernatant was collected, and its absorbance at 540 nm was recorded with a micro-plate spectrophotometer. A higher absorbance at 540 nm means stronger CHI activity. In this research, we defined one unit of CHI activity as a change of 0.1 OD per minute per gram FW at 540 nm. The results are presented as U·min^−1^·g^−1^ FW.

### 3.9. Effect of Oligochitosan DP5 on the Accumulation of Total Phenolics in Z. bungeanum

Phenolic compounds have been widely reported as one of the numerous antimicrobial compounds in plant tissues [63,81]. The accumulation of phenolic compounds in plants can be enhanced by elicitors to improve the resistance of plants to pathogen infection [27]. In this research, the accumulation of total phenolics in the twigs of *Z. bungeanum* elicited by DP5 was investigated. The twigs of *Z. bungeanum* were treated with DP5 as described in Section 3.8. The concentrations of DP5 applied in this experiment were 0.05, 0.1, and 0.2 mg/mL. The twigs were collected on days 2, 4, 6, 8, and 10 after treatment for extraction of total phenolics.

Total phenolics were extracted by ethanol as described previously with minor modifications [82]. Each sample (0.5 g, FW) was homogenized in 5 mL of 60% ethanol. The extraction mixture was treated ultrasonically for 30 min and then centrifuged at 10,000 rpm at 4 °C for 20 min. The supernatants were transferred into new 1.5-mL vials to measure total phenolics by the colorimetric method.

Measurements of total phenolics were conducted using the Folin–Ciocalteu method, and Gallic acid was taken as the standard [83,84]. For each reaction, 100 µL of Folin–Ciocalteu reagent (1 N) was mixed with 50 µL of sample solution. After adequate mixing, the reaction solution was held at room temperature for 5 min. Then, a 100-μL aliquot of Na_2_CO_3_ solution (10%) was added to the reaction solution, mixed well, and held at 25 °C for 2 h. The maximum absorption wavelength was determined by spectrum scanning at 765 nm. The standard curve was established, and the contents of total phenolics in samples were calculated. The Gallic acid equivalent (GAE), expressed in milligrams per gram FW (GAE·mg·g^−1^ FW) of sample, was used as an indicator of the total phenolic content in *Z. bungeanum* twigs [48].

### 3.10. Statistical Analysis

All results are presented as mean values and standard deviations (SD). The experimental data were submitted to an analysis of variance to detect significant differences using PROC ANOVA of SAS version 8.2 (SAS Institute, Cary, NC, USA).

## 4. Conclusions

In the present study, chitosan and oligochitosan were prepared from pathogen *F. sambucinum*, and their effects on infection of *Z. bungeanum* stems were investigated. Results showed that oligochitosan inhibited the infection of the pathogen, and the oligochitosan fraction with a DP between 5 and 6 showed the optimal effect. Oligochitosan DP5 was purified from the DP5–6 fraction, and it was characterized structurally using ESI-MS, FT-IR spectroscopy, and NMR spectroscopy. Oligochitosan DP5 showed significant inhibition on infection of host plants stems by pathogenic fungi. Further investigation of the mechanism underlying this effect demonstrated that oligochitosan DP5 increased the activities of defensive enzymes and accumulation of total phenolics in the host *Z. bungeanum*. These results suggest that oligochitosan from pathogenic fungi can mediate the process of pathogen infection of host plants as an elicitor, which will be useful for further exploration of the interactions between *F. sambucinum* and *Z. bungeanum*.

## Abbreviations

ESI-MS	Electrospray ionization mass spectrometry
FT-IR	Fourier transform infrared spectroscopy
NMR	Nuclear magnetic resonance
DA	Degree of acetylation
DP	Degree of polymerization
PAL	Phenylalanine ammonia lyase
PPO	Polyphenoloxidase
POD	Peroxidase
CHI	Chitinase
CCH	Crude chitosan
TOCH	Total oligochitosan
DCH	Deacetyated chitosan
DP < 5	Oligosaccharide fraction with a degree of polymerization less than five
DP5–6	Oligosaccharide fraction with a degree of polymerization between five and six
DP7–9	Oligosaccharide fraction with a degree of polymerization between seven and nine
DP > 9	Oligosaccharide fraction with a degree of polymerization greater than nine
DP5	Oligochitosan with a degree of polymerization of five
DEPT-135	Distortionless enhancement by polarization transfer (135°)
COSY	Correlation spectroscopy
NOESY	Rotating frame nuclear Overhauser effect spectroscopy
HSQC	Heteronuclear single-quantum correlation spectroscopy
GlcN	Glucosamine
